# Babies’ Sore Eyes

**Published:** 1875-04

**Authors:** Henry W. Williams

**Affiliations:** Professor of Ophthalmology at Harvard University


### BABIES' SORE EYES.

BY HENRY W. WILLIAMS, A.M., M.D.

*Professor of Ophthalmology at Harvard University.*

The accoucheur has scarcely begun to congratulate himself on the favorable progress of his case after delivery, when, in many instances, the appearance of ophthalmia in the new-born infant renews his anxieties. The suddenness of the attack, the severity of the symptoms, the delicate state of the mother and child—making it impossible in most cases to have other advice than that of the attending physician—and the immediate and obvious consequences of his skill or the want of it; these conditions combine to render such cases of grave importance.

From some cause, this disease has seemed to be more than usually frequent and virulent during the last summer and autumn, and I have been urged to say something in the *Journal* about it and its management.

No case should be neglected, when there is even a slight discharge from the eyes of a young infant; a mild form of conjunctivitis, however, is often met with, marked by slight redness of the lining of the lids and a little mucus secretion, which requires only frequent cleansing of the eyes with tepid water, and the use of simple ointment along the edges of the lids to prevent their adhesion at night; or, at most, the putting into the eyes, three times a day, a few drops of a solution of two grains of alum or four grains of borax in on ounce of water. These are the cases in which nurses think they accomplish such wonders by spirting into the eyes a stream of breast-milk: a waste of valuable material, but a procedure which does no other harm than to render the nurses self-confident, and to lead them to fatal reliance on the same means in cases of the more severe form of disease. This mild inflammation is apparently often caused by strong soap, or other acrid or irritating substances, rubbed into the eyes at the first cleansing of the child; cold and dampness are also causes. The same agencies may sometimes induce the more virulent disease which is the subject of this paper; but it is probably most often due to infection of the eyes, during birth, from vaginal or urethral secretions. This is made probable by the limitation of the time within which the first symptoms appear; for if the severer form of disease were often produced by the action of external irritants, it would show itself at various periods, as a result of the continued carelessness of mothers and nurses, whereas it seldom begins later than ten days after birth, usually much sooner.

The form of purulent conjunctivitis known as ophthalmia neonatorum, or ophthalmia of new-born infants, generally begins from the third to the sixth day after birth, a slight red streak on the skin along the middle of the upper lid being sometimes observed as a premonitory symptom before any discharge from the eyes is noticed. If the lid is drawn open, its lining is seen to be red and velvety, and a slight mucus secretion is found. In a few hours the lid may become enormously swollen and livid, the upper lid sometimes completely overlapping the lower, and resting upon the cheek. The conjunctiva lining the lid becomes greatly tumefied and its surface granulated, and inspection of the eye becomes impossible without the aid of an elevator. When by the help of this instrument the eye is seen, the conjunctiva of the eyeball is found to be in a condition similar to that of the inside of the lids. The secretion from the conjunctiva rapidly assumes a purulent character, and the quantity is very large, a teaspoonful perhaps accumulating in an hour's time. If this condition is not soon changed for the better, the defective nutrition, the pressure of the swollen lids, and maceration in the unhealthy secretion, cause haziness of the cornea, and then ulceration and perforation; followed usually by hernia of the iris, and perhaps loss of vision.

The opposite and equally fatal errors of treatment are unhappily prevalent. On the one hand, nurses frequently regard babies' sore eyes as a slight matter, and neglect to call the attention of the physician to the early symptoms, relying on the breast-milk as an infallible cure. Then, when the increased swelling of the lids makes the use of this means impossible, they are too often ready to apply an alum curd or a poultice "to draw the inflammation," thus greatly increasing the danger of ulceration or sloughing of the cornea. On the other hand, the physician, unfamiliar with these cases, and alarmed at the intensity and duration of the symptoms, feels that the latter must be subdued by active treatment, and may employ caustics or stimulants adapted to disease of the same tissues in adults, but not well borne by the infantile subject.

Of all curative means, the *most important is constant cleansing of the eyes*. This should be repeated according to the amount of the discharge, every two hours, every hour, or even every half hour during the day, and once or twice at least at night, until the diminished secretion and lessened thickness of the lids allow of a less frequent repetition. The lids may be opened with the fingers of both hands by the nurse, whilst another person pours in tepid water from a spoon or sponge. If the lids are greatly swollen, this becomes impossible, and a syringe must be used, which should be perfectly clean, and have a smooth and not too sharp point. Its nozzle is to be gently passed under the edge of the upper lid, and the contents injected so as thoroughly to wash out the palpebral cavity. This must be done often, as already advised, for it must be borne in mind that the continuous soaking of the cornea in the copious purulent discharge seems to soften its texture and prepare the way for ulceration. Special care should he taken, in cold weather, to make the water so warm that the child may have no shock, and thus to avoid its crying, as the thickened lids are often everted when the child cries. Should this eversion occur, the lids are to be replaced as gently as possible with the fingers. A little simple ointment should be used along the edges of the lids, when the child sleeps, to prevent agglutination and give opportunity for the free escape of the discharges; as also to protect the external skin from excoriation.

If these means are gently used, the child is not much disturbed, and soon falls to sleep after them. These measures for securing cleanliness appear to be sufficient for the cure of many even severe cases; but I think it safer, where the symptoms are formidable, to alternate with the injections of water the use of a mild astringent, as, for instance, a solution of five grains of alum in an ounce of water. This should be applied in the same way, and should be warmed if necessary. A solution of crystals of borax, of the same strength, may also be used. These are the best collyria for these cases; but a solution of sulphate of zinc, a fourth or a half of a grain in an ounce of water, may sometimes be serviceable. Any *strong* astringent solutions, or *any* solutions of nitrate of silver, acetate of lead, or corrosive sublimate; the introduction beneath the lids of mercurial or nitrate of silver ointments; the application of the crayon of nitrate of silver, pure or mitigated with nitrate of potash, or of the crayon of sulphate of copper: all these *should be avoided.* Cases may perhaps do well where these have been employed, especially if great care has at the same time been taken as regards cleanliness of the eyes; but they are dangerous remedies. Moreover, they sometimes evidently cause agonizing pain; and there is great risk that the mother, unable to bear the dreadful sight of her infant's sufferings, may refuse, unless the physician has established the strongest hold upon her confidence, to continue so harsh a treatment, and may place the child probably in less skillful hands, though blaming the doctor if the eyes are lost.

The condition of the cornea must be closely watched, and the lids must be raised for this purpose, by means of an elevator. If unprovided with such an instrument, the physician may form one by bending the end of the handle of a spoon, with which he can draw up the lid; or he may perhaps effect his object by using a broad hair-pin, bending the rounded end in the same way. Any central cloudiness or ulceration of the cornea would indicate the use of a drop of a solution of sulphate of atropia, two grains to an ounce of water, put into the eye once daily, or oftener, and continued while any cloudiness remains. Should perforation of the cornea take place, hernia of the iris may perhaps be prevented by its use, and if the opening is small and is promptly healed, good vision may be preserved. The physician should not relax his vigilance until the symptoms are much improved, as the cornea sometimes yields unexpectedly, under the effects of the long continuance of the disease, even in its later stages and after its force is apparently spent.

Every pains should be taken to secure good nutrition for the child. Without exposing it to the cold, the air of the room should be renewed. The light should be moderated, so that the child may open its lids when they are not too much swollen, and thus permit the discharge of the secretions. The child will not open its eyes if the room is too light or too dark.

The prognosis of this affection is favorable, even in the severest cases, if treated promptly and diligently from the outset; and I once more urge use of the simpler remedies as unquestionably the best. But if ulceration or a sloughy condition of the cornea is already present when treatment is begun, the result is often unfavorable, whatever means may be employed. Yet we need not wholly despair even where these conditions exist, as the eye will sometimes recover with at least partial vision.

A most important part of the physician's duty is to take every precaution against contagion. A minute particle of the morbid secretion may convey the disease to the eye of a healthy person. The attendant should therefore direct the thorough cleansing or destruction of all articles soiled with the purulent discharge; great care in using the syringe, so that no drop of the injection may be thrown back from beneath the lids into the eyes of the nurse; and immediate washing of the hands whenever they have touched the sore eyes or anything contaminated by them.—*Boston Medical and Surgical Journal.*

